# Risk factor analysis of childhood and adolescent thyroid cancer mediastinal lymph node and distant metastasis

**DOI:** 10.3389/fonc.2025.1705168

**Published:** 2025-11-21

**Authors:** Ruirui Zhang, Mengjin Sun, Siyue Ding, Jinling Niu, Shuhui Wang, Qian Li

**Affiliations:** Department of Ultrasound, The Affiliated Cancer Hospital of Zhengzhou University & Henan Cancer Hospital, Zhengzhou, China

**Keywords:** distant metastasis, thyroid cancer, children, ultrasonography, lymph node metastasis

## Abstract

**Objective:**

The aim of this study is to explore the risk factors related to mediastinal lymph node metastasis (MLNM) and distant metastasis (DM) in children and adolescents with thyroid cancer.

**Method:**

This is a retrospective analysis of children and adolescent thyroid cancer patients admitted to our hospital from January 2015 to October 2022. The clinical and pathological data, and the preoperative ultrasound features of the patients were statistically analyzed.

**Results:**

This study included a total of 67 patients, consisting of 20 males and 47 females. Among them, 14 cases (20.9%) had mediastinal lymph node metastasis and 21 cases (31.3%) had distant metastasis. The univariate analysis showed that gender, the central metastatic lymph node ratio (CCLNR), and DM were all correlated in MLNM. The nodule morphology, location, the CCLNR, and MLNM were identified as related factors in DM. The multivariate regression results showed that gender and DM were independent MLNM risk factors. Furthermore, the lesion morphology and the CCLNR were identified as independent DM risk factors.

**Conclusion:**

The larger the CCLNR ratio, the higher the likelihood of the development MLNM and DM in children and adolescent thyroid cancer patients. A CCLNR > 0.79 as an evaluation index can indicate that children and adolescents with thyroid cancer are at higher risk of developing MLNM and DM.

## 1 Introduction

The global incidence of thyroid cancer has markedly risen in the last two decades, notably among children and adolescents (1, 2). Although the occurrence of thyroid nodules in this demographic is relatively lower (approximately 1.5% to 3%) compared to adults, the malignancy rate is disproportionately higher and ranges from 18% to 50%, with a recurrence risk as high as 39% (2, 3). Furthermore, the distant metastasis (DM) incidence in this age group surpasses that of adults (4–6).

The majority (approximately 91.7%) of thyroid malignancies in children and adolescents consists of differentiated thyroid cancer (DTC). This encompasses papillary thyroid cancer (PTC), follicular thyroid carcinoma (FTC), and poorly differentiated thyroid cancer (P-DTC) (4).

Important indicators of advanced cancer are lymph node metastasis and distant metastasis. Many scholars believe that mediastinal lymph node metastasis (MLNM) and DM are closely related to the poor prognosis of children and adolescent patients. Hence, thyroid cancer management in children and adolescents requires early identification of tumors at risk of MLNM and/or DM, which is critically significant in guiding clinical decision-making.

## 2 Materials and methods

### 2.1 Research object

**Figure 1:**
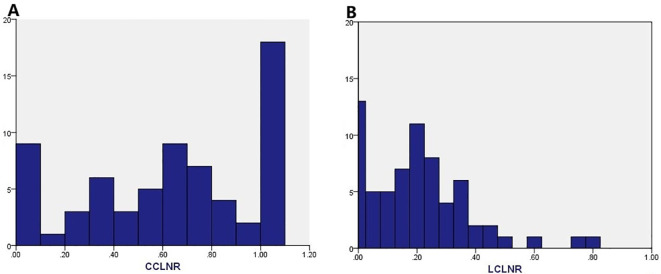
Histograms depicting the number of **(A)** CCLNR; **(B)** LCLNR.

A retrospective analysis was conducted on pediatric and adolescent thyroid cancer patients admitted to the Affiliated Cancer Hospital of Zhengzhou University (i.e., Henan Cancer Hospital) between January 2015 and October 2022. A total of 67 cases were included, comprising 19 males and 48 females aged 6 to 18 years (mean age: 14.55 ± 3.09). The patients’ baseline features are shown in **Figure 1**.

The inclusion criteria were as follows: 1) age ≤18 years old; 2) confirmed postoperative pathology of thyroid cancer; 3) a preoperative ultrasound, preoperative and postoperative CT/MR, or SPECT-CT examination was performed in our hospital; and 4) the patient had underwent a total thyroidectomy and bilateral central neck dissection/modified radical neck dissection (unilateral or bilateral).

The exclusion criteria were as follows: 1) patients with incomplete clinical data, 2) patients with previous thyroid surgery treatments, 3) patients with other malignant tumors, and 4) patients with a history of head and neck radiation.

### 2.2 Instruments and methods

Imaging examinations: Patients were positioned supine with the neck fully exposed for the ultrasound examinations. Scanning was performed on the bilateral glandular lobes, anterior cervical area, and lateral cervical area. All patient images were re-evaluated by an ultrasound physician with over five years of experience who was blinded to the patient’s pathological findings. The ultrasound examinations covered only cervical levels I–VI. The mediastinal area was defined as the region extending from the upper edge of the sternum to the upper edge of the aortic arch, which could not be visualized using ultrasound. Before surgery, patients in whom multiple lymph node metastases were detected on ultrasound were required to undergo chest computed tomography (CT) or magnetic resonance imaging (MRI) to evaluate the presence of mediastinum and pulmonary metastases. Postoperatively, SPECT-CT examination was performed to evaluate the whole body.

Surgery: The surgical scope for each patient was determined based on the patient’s preoperative examination. If the preoperative examination showed that the lesion was confined within the thyroid lobe with no abnormal lymph nodes, an ipsilateral central compartment dissection was performed. For cases with invasion of the surrounding tissues or abnormal central compartment lymph nodes, a bilateral central compartment lymph node dissection was performed. If abnormal lymph nodes were detected in the lateral cervical compartment, dissection of both the central and lateral cervical compartments was conducted. ^131^I Therapy: For the patients stratified as intermediate or high risk according to the American Thyroid Association (ATA) risk classification, a thyroid-stimulating hormone (TSH)-stimulated Tg test and Diagnostic Whole-Body Scan (DxWBS) were performed within 12 weeks after surgery to determine the need for ^131^I therapy. Due to differences in body sizes and iodine clearance rates between children and adults, the dosages for all pediatric thyroid cancer patients undergoing radioiodine therapy were calculated by a nuclear medicine specialist experienced in pediatric dosing. All patients received ^131^I therapy only after obtaining informed consent from their guardians.

The pretherapy assessment criteria were as follows: 1) radioiodine-avid distant metastases, 2) unresectable local radioiodine-avid residual disease or metastatic lymph nodes, 3) DxWBS-negative with a stimulated Tg (s-Tg) level ≥ 10 ng/mL, and 4) DxWBS-negative with a stimulated Tg (s-Tg) level between 2 ng/mL and 10 ng/mL. ^131^I therapy may have also been considered in these cases after weighing the benefits and risks.

Histopathological examination: The pathologists diagnosed thyroid cancer, membrane invasion, surrounding tissue invasion, vascular invasion, lymph node metastasis, affected lymph node count, and the presence of mutations in the BRAF gene. The metastatic lymph nodes were categorized as central compartment lymph node metastasis (CCLNM) or lateral compartment lymph node metastasis (LCLNM) based on the dissection location and pathological findings. The lymph node ratio (LNR) was calculated as the ratio of the positive lymph nodes to the total number of nodes dissected.

MLNM and DM evaluation: CT or MRI scans were conducted pre-surgery, and some patients underwent systemic SPECT-CT imaging within a month post-surgery. Surgical pathology or imaging results were used to analyze the presence of mediastinal lymph node metastasis or distant metastasis.

### 2.3 Statistical analysis

SPSS 23.0 was used for the data analysis. The observational data primarily consisted of enumeration data. Data that conform to the normal distribution were expressed as mean ± standard deviation, while those that did not conformed to the normal distribution were expressed as a median and quartile. The correlation analyses involved the utilization of a bilateral Fisher’s exact test or chi-square test for the categorical variables. A point binary correlation analysis was conducted for both the categorical variables and continuous variables. A multivariate correlation analysis was conducted using an unconditional logistic regression and the forward linear regression (LR) method. A bilateral significance test was performed, and the statistical significance was set at *P* < 0.05.

## 3 Results

Between 2015 and 2022, 96 pediatric patients with thyroid disease received treatment at our institution. A total of 29 cases were excluded from the study due to incomplete clinical data. A total of 67 patients met the inclusion criteria, and they consisted of 48 females (71.6%) and 19 males (28.4%). Among them, 50 cases (74.5%) were classified as the classic thyroid carcinoma (C-PTC), 11 cases (16.4%) as the follicular variant of papillary thyroid carcinoma (FV-PTC), and 6 cases as other pathological types that included 3 cases of diffuse sclerosis variant papillary thyroid carcinoma (DSV-PTC), 2 cases of PTC with squamous metaplasia, and 1 case of poorly differentiated thyroid carcinoma (P-DTC). The follow-up period for patients ranged from 3 months to 8 years. The baseline characteristics of the patients are presented in **Table 1**.

**Table 1:** Baseline features of the 67 patients.

		Average ± SD	Patients *n*(%)
Gender			
	Male		19(28.4%)
	Female		48(71.6%)
Age		14.55 ± 3.09	
Pathological type			
	C-PTC		50(74.6%)
	FV-PTC		11(16.4%)
	DSV-PTC		3(4.5%)
	PTC with squamous metaplasia		2(3.0%)
	P-DTC		1(1.5%)
Surgery type			
	Unilateral thyroidectomy		2(3.0%)
	Total thyroidectomy		65(97.0%)
	Unilateral central neck lymph node dissection		2(3.0%)
	Bilateral central neck lymph node dissection		65(97.0%)
	Unilateral lateral neck lymph node dissection		57(85.1%)
	Bilateral lateral neck lymph node dissection		17(25.4%)
T-stage			
	T1		29(43.3%)
	T1a		7(10.4%)
	T1b		22(32.8%)
	T2		27(40.3%)
	T3		9(13.4%)
	T4		2(3.0%)
N-stage			
	N0		9(13.4%)
	N1a		5(7.5%)
	N1b		53(79.1%)
M-stage			
	M0		54(80.6%)
	M1		13(19.4%)
ATA class risk			
	Low		9(13.4%)
	Intermediate		36(53.7%)
	High		22(32.9%)
^131^I therapy			49(73.1%)

A total of 49 patients underwent ^131^I therapy, with ages ranging from 6 to 18 years. Evaluations were conducted within 3 months postoperatively, and all patients that required radioiodine treatment received their initial therapy within 4 months after surgery. The administered activity per treatment session ranged from 50 to 150 mCi, with cumulative total doses ranging from 50 to 4950 mCi. The number of treatment sessions varied from one to nine, with treatment intervals ranging between 4 and 10 months.

A total of 61 cases (91.0%) of LNM occurred. These included 58 (86.65%) CCLNM cases and 56 LCLNM cases (80.6%). The median of the CCLNM cases was four (2, 8), and the median of the LCLNM cases was five (2, 11). The CCLNR was 0.67 (0.36, 1.00), and the LCLNR was 0.18 (0.07, 0.29), as shown in **Figure 1**.

There were a total of 14 MLNM patients (20.9%): 8 patients were detected prior to the MLNM surgery, 4 patients were detected 3 months after surgery, 1 patient was detected 6 months after surgery, and 1 patient was detected 2 years after surgery. Two patients underwent superior mediastinal lymph node dissection.

There were a total of 21 DM patients (31.3%), including 19 with lung metastasis and two with bone metastasis. A total of 13 patients were diagnosed before surgery, six patients were diagnosed 3 months after surgery, one patient was diagnosed 12 months after surgery, and one patient was diagnosed 18 months after surgery.

**Table 2** shows the various patient characteristics and the results of the univariate analysis. Gender, multifocality, the central compartment lymph node ratio (CCLNR), and DM were associated with MLNM. The nodule morphology, location, MLNM, and the CCLNR were related factors for DM (*P* < 0.05). **Table 3** shows the results of the multiple logistic regression. Gender and DM were independent risk factors for MLNM, and lesion morphology and CCLNR were independent risk factors for DM (*P* < 0.05).

**Table 2:** Characteristics of the study patients and univariate analysis of predictors.

Characteristics	Totally (%)	MLNM		*P*	DM		*P*
		Positive	Negative		Positive	Negative	
Gender(Male/Female)	19(28.4)/48(71.6)	9/5	10/43	0.002	7/14	12/34	0.569
Pathological type(C-PTC/FV-PTC/Other)	50(74.6)/11(16.4)/6(9.0)	14(10/4/0)	53(40/7/6)	0.254	21(15/6/0)	46(35/5/6)	0.064
Capsule invasion(Positive/Negative)	30(44.8)/37(55.2)	7/7	23/30	0.766	11/10	19/27	0.437
Surrounding tissue infiltration(Positive/Negative)	14(20.9)/53(79.1)	5/9	9/44	0.149	7/14	7/39	0.112
Vessel invasion(Positive/Negative)	8(11.9)/59(88.0)	0/14	8/45	0.189	1/20	7/39	0.419
Multifocality(Single nodule/multiple nodules)	39(58.2)/28(37.3)	4/10	35/18	0.016	10/11	29/17	0.290
Braf(Positive/Negative/Unkown)	15(22.4)/33(49.3)/19(28.4)	1/10	14/23	0.136	2/15	13/18	0.050
LNM(Positive/Negative)	61(91.0)/6(9.0)	14/0	47/6	0.330	20/1	41/5	0.657
CCLNM(Positive/Negative)	58(86.6)/9(13.4)	14/0	44/9	0.186	19/2	39/7	0.709
LCLNM(Positive/Negative)	54(80.6)/13(19.4)	14/0	40/13	0.055	18/3	36/10	0.531
Tumor size (mm)(≤10/10~20/20~30/≥30)	7(10.3)/18(26.9)/18(26.9)/24(35.9)	0/3/5/6	7/15/13/18	0.467	1/6/8/6	6/12/10/18	0.435
Position(Non diffuse /Diffuse)	43(64.2)/24(35.9)	9/5	34/19	1.000	16/5	27/19	0.185
Calcifications(Speckled/Coarse/None)	49(73.1)/9(13.4)/9(13.4)	12/2/0	37/7/9	0.335	17/2/2	32/7/7	0.648
Taller-than-wide shape(<1/≥1)	62(92.4)/5(7.6)	14/0	48/5	0.355	20/1	42/4	0.670
Margins (Smooth/Irregular)	9(13.4)/58(86.6)	0/14	9/44	0.186	2/19	7/39	0.709
Echogenicity (Hypoechoic/Hyperechoic or isoechoic)	54(80.6)/13(19.4)	11/3	43/10	1.000	16/5	38/8	0.740
Shape(rule/Irregular)	14(20.9)/53(79.1)	2/12	12/41	0.716	1/20	13/33	0.049
LNR				0.308			0.103
CCLNR				0.005			0.002
LCLNR				0.121			0.067

**Table 3:** Multivariate analyses of risk factors of MLNM and DM.

	Odds ratio	Odds ratio (95%CI)	*P*
MLNM			
Gender	0.152	0.038-0.608	0.008
CCLNR	5.887	1.446-23.963	0.013
Multifocality			0.077
DM			
Shape	16.970	1.691-170.313	0.016
CCLNR	17.508	4.438-69.067	<0.001

**Figure 2:**
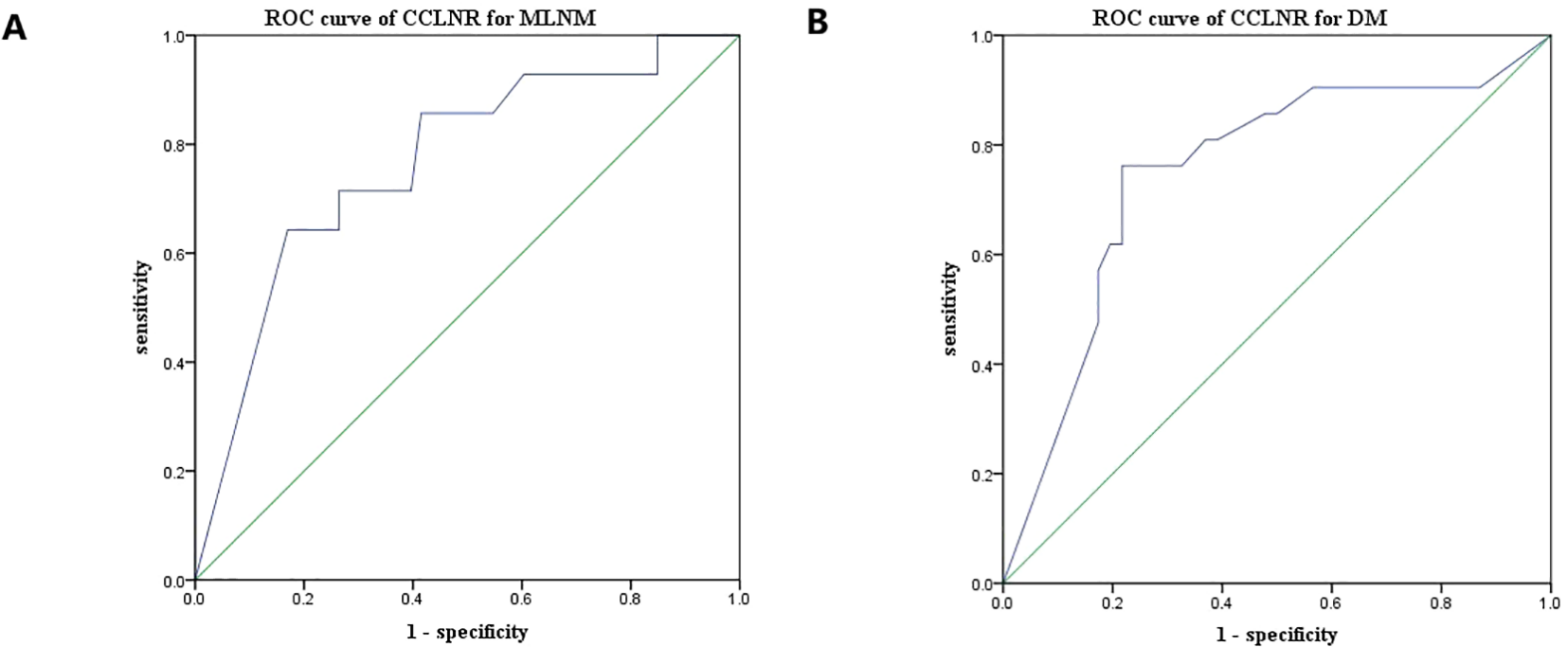
ROC curves of CCLNR for evaluating MLNM **(A)** and DM **(B)**.

For DM, the cutoff value of CCLNR was 0.775 (*P* < 0.05), and the area under the curve was 0.751. For MLNM, the cutoff value of CCLNR was 0.790 (*P* < 0.05), and the area under the curve was 0.767, as shown in **Figure 2**.

## 4 Discussion

The ATA published management guidelines for thyroid nodules in children and adolescents in 2015. They classified the severity of thyroid cancer cases in this age group into three risk categories (7). Patients in the low-risk group do not experience lymph node metastasis, whereas those in the intermediate-risk group present with only a limited number of central lymph node metastases. High-risk patients, characterized by extensive cervical lymph node metastases, MLNM, and/or DM, face the highest probabilities of recurrence, persistent disease, and adverse prognoses. MLNM and DM serve as crucial indicators of advanced cancer. Pediatric PTC patients exhibit a high prevalence of DM. While prior research has indicated that DM does not pose an immediate threat to the lives of children and adolescents, it significantly affects their quality of life and contributes to an elevated mortality risk (8).

Ultrasonography constitutes the foremost imaging modality for thyroid examination of patients in the low- and medium-risk categories, and it plays a pivotal role in distinguishing between benign and malignant lesions, as well as in diagnosing metastatic lymph nodes in the neck. However, ultrasound exhibits low sensitivity when detecting deep tissue lesions and offers limited comprehensive evaluation value for patients in high-risk groups. Consequently, it is still necessary to incorporate additional examinations to enhance patient condition assessments. Pediatric patients are more sensitive to radiation; therefore, CT scans are not recommended for those in whom ultrasound examination does not reveal extensive lymph node metastasis. While positron emission tomography-computed tomography is essential to identify mediastinal and distant metastases, its utility is restricted prior to thyroidectomy due to physiological tracer uptake by thyroid tissue. Therefore, indicators are required to improve risk stratification for MLNM and DM in children and adolescent thyroid cancers.

The findings of this study showed males, multifocal tumors, and the CCLNR were related MLNM risk factors, with males and the CCLNR identified as independent risk factors. These results agreed with those of previous research outcomes (8–11). Thyroid cancer commonly emerges during adolescence when patients are primarily between 15 and 18 years of age, a trend reflected in study and others (3, 13). Male patients often exhibit higher tumor staging, increased recurrence rates, and poorer prognoses compared to females (6, 14, 15). Similarly, our study confirmed that being male is an independent MLNM risk factor, indicating the heightened invasiveness. Multifocal lesions showed an association with MLNM, but this lacked predictability, which agreed with prior findings (16).

The findings of this study revealed that morphology and CCLNR are independent risk factors for DM. Prior research on cervical lymph node metastasis had established a link between nodule morphology and aggressiveness, and our study similarly confirmed its predictive capacity for DM. An irregular thyroid nodule morphology is characterized by an uneven contour with jagged, spiculated, or angulated margins. It demonstrates features of infiltrative growth into surrounding tissues, and this is a radiographic manifestation of tumor aggressiveness. Previous studies have indicated that an irregular nodule shape is associated with malignancy and possesses a certain predictive value for lymph node metastasis (17). The results of this study revealed a correlation between irregular morphology and DM that may be attributed to the higher aggressiveness of tumors with irregular shapes. However, current research on the relationship between nodule morphology and distant metastasis in pediatric and adolescent thyroid cancer remains limited, with small sample sizes and inconclusive findings (16, 18). Further investigation is required to explore this correlation.

While previous research has suggested that microcalcifications, solid composition, and extrathyroidal extension are associated with malignant thyroid nodules and cervical lymph node metastasis in children, this study did not find significant correlations between these features and MLNM or DM (19, 20). Studies by some scholars have indicated in the case of pediatric and adolescent patients, those with tumors smaller than 1 cm in diameter exhibited a significantly higher incidence of lymph node metastasis compared to adults, with the risk of lymph node metastasis not escalating with tumor size. The results of our study concurred that lesion size is not closely associated with MLNM and DM. For pediatric and adolescent patients, the extent of surgery should not be determined solely by tumor size, as even smaller lesions can potentially lead to advanced disease stages.

The CCLNR is a strongly associated risk factor for both MLNM and DM. The areas under the receiver operating characteristic curves plotted from the respective data for both were greater than 0.75, indicating a high diagnostic value. The cut-off values for the two were also relatively close, at 0.78 and 0.79. Detailed lymph node information included counts and ratios aids in disease staging evaluations and recurrence risks (10–12). The initial surgical approach significantly impacts underage patient survival rates. Adequate lymph node dissection is crucial for accurate disease assessment and to minimize recurrence risks. Currently, the number of lymph nodes to be cleared lacks a unified standard; hence, an evaluation of the surgical effectiveness, disease staging, and prognosis through the lymph node ratio becomes more pragmatic. The findings of this study indicated that a CCLNR greater than 0.79 served as an independent risk factor for MLNM, while a CCLNR that was greater than 0.78 was an independent risk factor for DM. Both can be employed as indicators to assess prognostic risks.

In this study, 71.4% (10/14) of MLNM patients had DM, and 47.6% (10/21) of DM patients had MLNM. These results indicated that MLNM patients are prone to develop DM. The statistical results showed a high correlation between MLNM and DM. The incidence of secondary DM in patients with MLNM was much greater than that in patients without MLNM.

It was also found that the relevant predictive factors primarily depended on postoperative pathological characteristics. The results indicated that a preoperative ultrasound examination alone cannot optimize treatment plans. For patients who exhibit specific manifestations in postoperative pathology, it is necessary to improve monitoring of distant metastasis in subsequent follow-up examinations, such as increasing the frequency and examination items of follow-up examinations. This can better monitor disease progression and improve patient quality of life.

All data originated from tertiary medical centers, where the malignancy rates of patients were high and there were many advanced cases. The predictive factors summarized are also applicable to secondary diagnosis and treatment centers. A limitation of this study is its small sample size. Thus, the next step is to expand the sample size and conduct multi-center research.

## 5 Conclusion

Thyroid cancer in children and adolescents often involves high rates of lymph node and distant metastases. The LNR is a valuable indicator used to assess disease staging. Male patients should remain vigilant against MLNM. In addition, individuals with irregular nodule morphologies and a postoperative pathological CCLNR that exceeds 0.78 face a heightened risk of distant metastasis. Consequently, a more comprehensive postoperative follow-up is crucial for the early detection of metastatic lesions and the development of suitable treatment plans.

## Data Availability

The raw data supporting the conclusions of this article will be made available by the authors, without undue reservation.
